# Laparoscopic heminephrectomy of a horseshoe kidney with giant renal cell carcinoma: A case report

**DOI:** 10.3892/ol.2014.2455

**Published:** 2014-08-19

**Authors:** XIAOLONG QI, FENG LIU, QI ZHANG, DAHONG ZHANG

**Affiliations:** Department of Urology, Zhejiang Provincial People’s Hospital, Hangzhou, Zhejiang 310014, P.R. China

**Keywords:** horseshoe kidney, renal cell carcinoma, laparoscopic heminephrectomy

## Abstract

A 72-year-old male was diagnosed incidentally with a 7-cm renal tumor in the right moiety of a horseshoe kidney during a routine physical examination, and was treated with laparoscopic radical heminephrectomy. The surgical time was 153 min and the estimated blood loss was 150 ml. The patient was discharged on post-operative day eight with no complications. Computed tomography angiography is desirable prior to surgery for the evaluation of the anatomical variations of horseshoe kidneys, and a pre-operative understanding is necessary for achieving reliable vascular control. In conclusion, the present technique is safe and effective for this complex clinical condition.

## Introduction

Horseshoe kidneys are the most common renal fusion anomaly. Abnormal vasculature and the possibility of isthmusectomy are the primary issues that require attention when surgery is considered for renal cell carcinoma in horseshoe kidneys. To date, there have been few reports of renal cell carcinomas excised from horseshoe kidneys using the laparoscopic or hand-assisted laparoscopic radical heminephrectomy approaches (1–4). Therefore, the incidence, treatment and outcome of such remains unclear. The present study describes the case of a pure transperitoneal laparoscopic radical heminephrectomy for a large renal tumor in a horseshoe kidney. Written informed consent was obtained from the patient.

## Case report

### Case details

**Figure 1:**
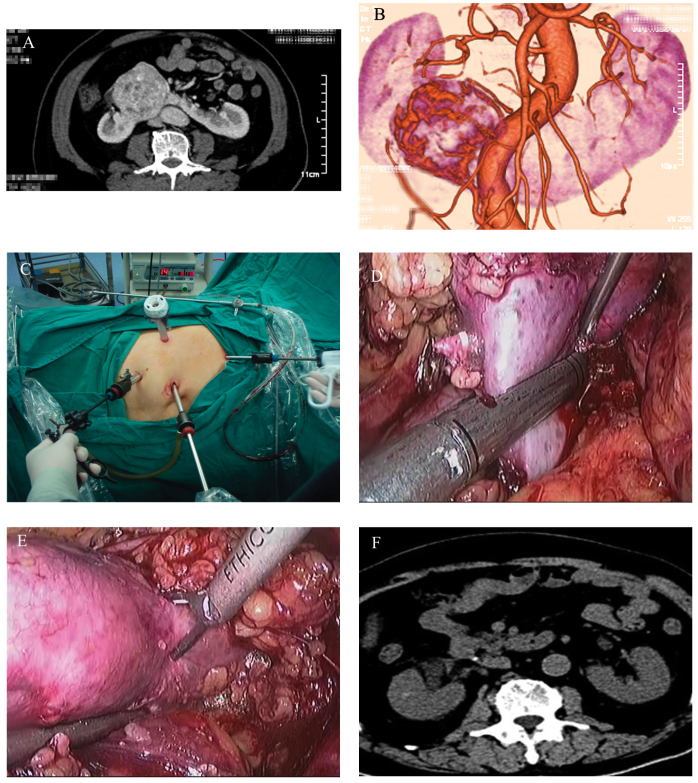
(A and B) Pre-operative computed tomography angiography (CTA). (C) The location of the port sites during surgery. (D) The ismthus was divided using the endoscopic stapler. (E) The tumor was excised with ultrasonic scissors. (F) CTA demonstrating no signs of disease relapse subsequent to an 18-month follow-up.

A solid renal tumor in the right moiety of a horseshoe kidney was incidentally detected in a 72-year-old male during a routine physical examination. Computed tomography angiography (CTA) identified a 7-cm enhancing mass supplied by three arteries in the right renal moiety (Fig. 1A and B). The results of the evaluation for metastases were negative. Following a careful explanation of the risks and benefits of alternative treatments, the patient elected to undergo a laparoscopic radical heminephrectomy.

### Surgical technique

Under general anesthesia, a 16F urethral catheter was inserted. The patient was placed in a 80° left lateral decubitus position and a transperitoneal access approach was used. A 14-mmHg pneumoperitoneum was established first. The positioning and trocar placements are shown in Fig. 1C. Mobilization of the ascending and transverse sections of the colon revealed the underlying kidney with a wide isthmus. Mobilization of the right side of the horseshoe kidney extending to the isthmus was carried out following the exposure of the inferior vena cava. The tumor was identified in the inferoanterior section of the right kidney. An Endo-GIA vascular stapler (Ref, 6TB45; 5-mm staple line, 3.5-mm staple leg length; six rows; Ethicon Endo-Surgery, Inc., Blue Ash, OH, USA) was used for the division of the isthmus (Fig. 1D), while the renal arteries and veins were secured with Hem-O-Lok clips (Teleflex Medical, Research Triangle Park, NC, USA). The tumor excision was performed using ultrasonic scissors (UltraCision; Ethicon Endo-Surgery, Inc.) (Fig. 1E). The renal tissue defect was repaired using Vicryl 1 sutures (SutureVCP358; Ethicon Endo-Surgery, Inc.). The excised tissue was removed with an Endo-bag (T Bag; Guangzhou TK Medical Instrument Co., Ltd., Guangzhou, China). Frozen sections of the tissue confirmed tumor-free borders. The surgical time was 153 min and the estimated blood loss was 150 ml. There were no immediate or delayed complications. Physical activity and oral intake were resumed on the day after surgery. The patient was discharged on post-operative day eight. Pathology revealed a pT2N0M0 grade 3 clear cell carcinoma of the kidney with free surgical margins. Following an 18-month follow-up, there was no disease relapse (Fig. 1F).

## Discussion

Hayakawa *et al* (5) reported the first practical application of laparoscopic partial nephrectomy using microwave coagulation for the heminephrectomy of a horseshoe kidney. The treatment of a tumor localized in a horseshoe kidney is challenging. The present study describes a case treated by transperitoneal radical heminephrectomy for the laparoscopic removal of a large tumor (7 cm; pT2) from a horseshoe kidney. Horseshoe kidneys have unique anatomical features that make surgery technically challenging. These include aberrant vasculature, abnormal kidney location and the renal isthmus. Pre-operative CTA is appropriate for determining the tumor size and location, and the extrarenal anatomy of the renal vessels.

The treatment of horseshoe kidneys by minimally invasive surgery using laparoscopy is rapidly becoming the leading most suitable surgical option. The advancement in laparoscopic instruments and techniques has led to the management of renal cell carcinoma in horseshoe kidneys. Transperitoneal laparoscopic heminephrectomy is an achievable and effective alternative to conventional management of horseshoe kidney tumors.

In conclusion, in the management of horseshoe kidneys with renal cell carcinoma, laparoscopic heminephrectomy has been demonstrated to be a valuable alternative treatment. The technique is a challenging approach and more experience is required prior to it becoming the standard of care. The radical heminephrectomy technique is useful in patients with horseshoe kidneys, as it provides the surgeon with a safe and efficient approach for the treatment of renal cell carcinoma in these patients.
